# The complete mitochondrial genomes of *Dactylella tenuis*, a fungus phylogenetically close to nematode-trapping fungus

**DOI:** 10.1080/23802359.2019.1644229

**Published:** 2019-09-04

**Authors:** Wen-Jun Li, Ze-Fen Yu

**Affiliations:** a State Key Laboratory for Conservation and Utilization of Bio-Resources in Yunnan, and Key Laboratory for Southwest Microbial Diversity of the Ministry of Education, Yunnan University, Kunming, P. R. China;; b School of Life Science, Yunnan University, Kunming, P. R. China

**Keywords:** Orbiliaceae, nematode-trapping fungus, phylogenetic analyses

## Abstract

In our study, we sequenced the complete mitochondrial genome of *Dactylella tenuis* and obtained the complete mitochondrial DNA sequence. This mitogenome is a typical circular molecule of 186,056 bp in length, which is rich in AT (73.79%), including 14 protein-coding genes, 24 transfer RNA genes, and 2 ribosomal RNA genes. Phylogenetic analysis showed the evolutionary relationship between *D. tenuis* and other species of nematode-trapping fungus.

Nematode-trapping fungus an is anamorphic state of Orbilia (Ascomycota, Orbiliomycetes, Orbiliales, Orbiliaceae), which capture nematodes using specialized vegetative hyphae called capturing device. Main capturing device includes adhesive net, adhesive knobs, adhesive columns, and constricting rings. The mitogenome of several species with the different capturing devices were reported (Zhou et al. 2018; Fang et al. 2019; Zhang and Yu 2019; Deng and Yu 2019a, 2019b). *Dactylella tenuis*, which does not form capturing device but is considered to be the ancestor of nematode-trapping fungus, its mitogenome has not been reported. Here, we reported the complete mitochondrial genome sequence of *D. tenuis* (CBS325.70) and its phylogenetic relationships to other nematode-trapping fungus based on concatenated amino acid sequences of 14 mitochondrial protein-coding genes.

The mitogenome was extracted from the whole genome sequence of a pure culture of strain CBS325.70. Mitogenome assembly was performed by the software SPAdes 3.9.0 (Bankevich et al. 2012). The mitogenome was annotated using MFANNOT (http://megasun.bch.umontreal.ca/cgi-bin/mfannot/mfannotInterface.pl), and was manually adjusted and transfer RNA genes were identified using tRNAscan -SE1.2.1 (Lowe and Eddy 1997) with the default search mode. All ORFs were searched and identified by ORFFinder (https://www.ncbi.nlm.nih.gov/orffinder? tdsourcetag=s_pcqq_aiomsg).

The complete mitochondrial genome of *D. tenuis* (CBS 325.70) was 186,056 bp in length with GC content of 26.21%, which contains 14 mitochondrial protein-coding (PCGs) genes, 24 tRNA genes (tRNA) and 2 ribosomal RNA genes (rnl and rns). The 14 conserved protein-coding genes include nad1, nad2 (five introns), nad3 (two introns), nad4 (one introns), nad4L, nad5 (seven introns), nad6, cox1 (12 introns), cox2 (three introns), cox3 (eight introns), atp6 (one introns), atp8, atp9 (two introns), and cob (six introns). All tRNA genes are encoded on the sense strand. GenBank accession number is MK820634.

*Heterobasidion irregulare*, *Microbotryum lychnidisdioicae,* and *Pneumocystis carinii* were used as outgroup for tree rooting. The BI tree (Figure 1) showed that the nematode-trapping fungus evolved from *D. tenuis*, subsequently, constricting rings, adhesive columns, adhesive net, adhesive knobs formed successively.

**Figure 1.**
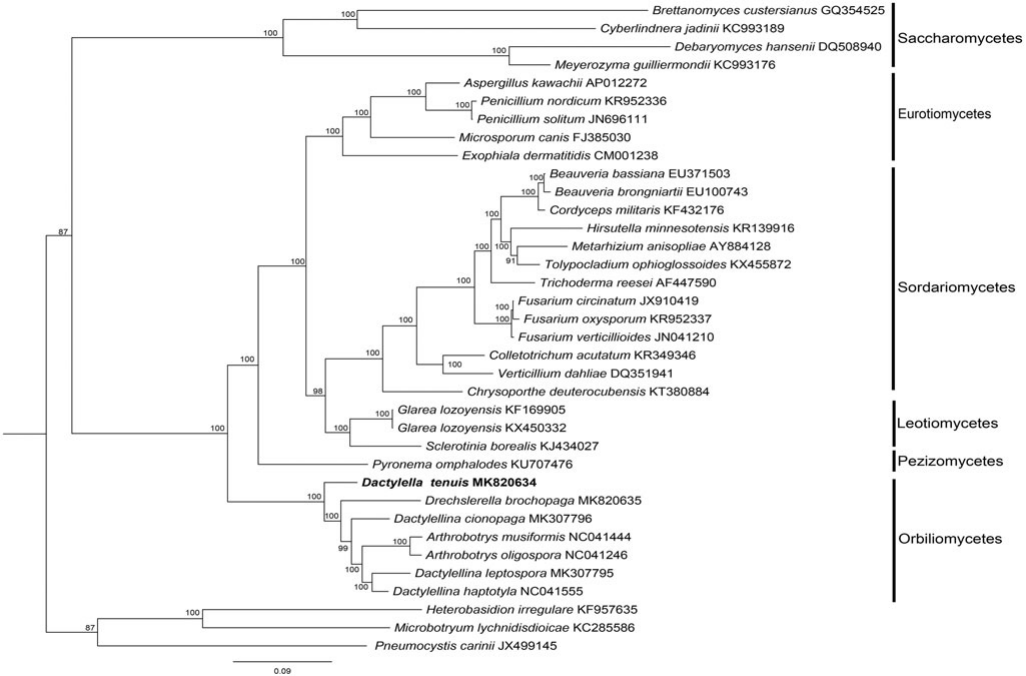
The phylogenetic tree of the *Dactylella tenuis* mitogenome and related organisms based on 14 conserved mitochondrial protein-coding genes (cox1, atp6, nad2, nad3, cox3, cob, nad4L, nad5, nad6, cox2, atp8, nad4, atp9, nad1). The conserved mitochondrial protein-coding genesis downloaded from GenBank and the phylogenic tree was generated using Bayesian inference (BI).
